# 

**Published:** 1910-11-19

**Authors:** 


### CONTENTS.

| | PAGE |
|---|---|
| **Urbs in Rure** | 215 |
| **Sunday Observance** | 216 |
| **Annotations—** Facial Asymmetry—The Cinematograph in Medicine | 217 |
| **Medical Opinion and Movement** | 218 |
| **Hospital Clinics—** Some Cases of Diabetes. By Nestor Tirard, M.D., F.R.C.P. | 221 |
| **Special Article—** Carbon Dioxide Snow | 222 |
| **Medicine—** Infective Endocarditis due to an Organism not hitherto described | 223 |
| **Surgery—** Environment and Operation | 225 |
| **The General Practitioner's Column—** The Value of Acetyl-Salicylic Acid in Certain Affections of the Eye | 226 |
| **Motoring Notes—** Some Points in the Choice of a Car—II. | 227 |
| **Orthopædics—** Pain in Some Foot Cases—III. | 228 |
| **News and Events of the Week** | 229 |
| **Obituary** | 230 |
| **Parliament and Publications** | 230 |
| **Appointments and Resignations** | 230 |
| **"The Hospital" Medical Book Supplement—No. XXXVI.** | 231 |
| **Editor's Letter-Box—** Charing Cross Hospital—An Appeal for the Queen's | 236 |
| **The Week's Work—** Operations in Children's Wards—North Staffordshire Infirmary—Its Complete Reorganisation—The Charing Cross Hospital Appeal—The Drug-room Beetle—This Week's Drug Market | 237 |
| **Special Institutional Articles—** The American Hospital Association | 239 |
| Public Grants for Voluntary Hospitals | 240 |
| **The Hospital World** | 241 |
| **Institutional Notes and News** | 243 |
| **Gifts and Bequests** | 244 |
| **Jottings** | 244 |

